# Importance of social capital at the workplace for return to work among women with a history of long-term sick leave: a cohort study

**DOI:** 10.1186/s12912-017-0234-2

**Published:** 2017-07-14

**Authors:** Ingela Rydström, Lotta Dalheim Englund, Lotta Dellve, Linda Ahlstrom

**Affiliations:** 1 Faculty of Caring Science, Work Life and Social Welfare, University of Borås, 501 90 Borås, Sweden; 2 Department of Sociology and Work Science, University of Gothenburg, Gothenburg, Sweden; 3 Health and Care Sciences, the Sahlgrenska Academy, University of Gothenburg, Gothenburg, Sweden

**Keywords:** Cohort, Sickness absence, Social attachment, Social capital, Vitality, Work ability, Quality of leadership

## Abstract

**Background:**

The workplace is an essential source of social capital for many people; it provides mutual support and gives meaning to life. However, few prospective studies have thoroughly investigated the importance of aspects of social capital in the workplace. The aim of this study was to investigate the associations between aspects of social capital (social support, sense of community, and quality of leadership) at the workplace, and work ability, working degree, and vitality among women with a history of long-term sick leave from human service organizations.

**Methods:**

A longitudinal cohort study was performed among women with a history of long-term sick leave. The study started in 2005, and the women were followed up at 6 months, 1 year, and 6 years using self-reported questionnaires (baseline *n* = 283). Linear mixed models were used for longitudinal analysis of the repeated measurements of prospective degree of work ability, working degree, and vitality. Analyses were performed with different models; the explanatory variables for each model were social support, sense of community, and quality of leadership and time.

**Results:**

Social capital in terms of quality of leadership (being good at solving conflicts and giving high priority to job satisfaction), sense of community (co-operation between colleagues) and social support (help and support from immediate superiors and colleagues) increased the women’s work ability score (WAS) as well as working degree over time. Additionally, social capital in terms of quality of leadership increased the women’s vitality score over time.

**Conclusions:**

A sustainable return-to-work process among individuals with a history of long-term sick leave, going in and out of work participation, could be supported with social support, good quality of leadership, and a sense of community at the workplace. The responsibility for the rehabilitation process can not be reduced to an individual problem, but ought to include all stakeholders involved in the process, such as managers, colleagues, health care services, and the social security agency.

## Background

The workplace is an essential source of social capital for many people; it provides mutual support and gives meaning to life. Apart from sleeping, paid work is the activity that occupies most of our time in adult life [1]. Some people do not share in the social capital that a job can bring, sometimes due to unemployment, but also, in other cases, due to long-term sick leave [2]. Few prospective studies have thoroughly investigated the importance of aspects of social capital in the workplace.

### Social capital and sustainable work situation

Social capital is one of the most important determinants for a human’s health and wellbeing [3]. According to Adler and Kwon, the concept of social capital can be understood as “the goodwill that is engendered by the fabric of social relations and that can be mobilized to facilitate action” [4]. Social capital can be seen as an “umbrella concept” covering key factors such as social support, trust, recognition, and reciprocity [4–7], and this concept seems to be an important aspect of relations [8].

According to Papastavrou et al. [9], research into social capital has followed one of three directions. The first direction corresponds to the managerial and work perspective, which, according to Read [10], illuminates the value of social relations at work, the leadership styles of managers, relationships between team members, and organizational commitment [11]. The second direction is concerned with social capital in relation to health and care; high social capital has been found to be a predictor of employee health [12–14].Moreover, important factors for the perception of wellbeing include social interactions, value and meaning at work, a good social atmosphere [15], being accepted, being respected, and receiving support from coworkers [13]. The third direction described by Papastavrou et al. [9] is social capital in relation to factors in the community or at home. By identifying the current attributes of nurses’ social capital, Read [10] formulated a definition of the concept of social capital: “nurses’ shared assets and ways of being and knowing that are evident in, and available through, nurses’ networks of social relationships at work”. Nurse managers can increase social capital at the workplace by improving communication, trust, and positive leadership at their practices; such a climate will allow workers to be more satisfied with and engaged in their jobs, and hence do their jobs more effectively.

Social capital with good social interactions at work has central importance for work-related health, and contributes to sustainable work organizations and increased work satisfaction among employees [14]. Having high social capital at the workplace is also an important factor in the process of return to work (RTW) [16, 17]. In addition, the social atmosphere at work plays an important role in generating sick leave [18]. Health impairments are higher among employees working in units with low social capital, compared to those in units with high social capital [19]. Social support at the workplace is important for RTW [20–22], and poor social support from one’s colleagues and manager can also contribute to sick leave [23]. Weak social support has been found to be a predictor of long-term sick leave [24]. Furthermore, social relations with coworkers and support from coworkers are predictive determinants for RTW after sick leave among women treated for breast cancer [25]. On the other hand, a recent review study found that there is inconclusive evidence for the effect of social support among individuals with a musculoskeletal disability, regarding physical, psychological and economic recovery, and the authors highlighted the need for further research [26].

Long-term sick leave is increasing among women in Sweden and in other European countries. Women have a higher rate of sickness absence than men, in terms of both frequency and number of spells of sick leave [27]. This brings great costs and suffering to society as well as to the individual. The effects of increased work demands and intensifications in organizations have also placed increased demands on individuals, particularly among women and at female-dominated work places [28]. Thus, there is great interest in developing strategies to promote a sustainable RTW. In Sweden, the sickness insurance system covers the entire working population. The principle is to provide compensation through sickness benefits when a worker has decreased work ability due to sickness or injury (i.e. medical conditions) and therefore is not able to work. A worker needs to lose at least 25% of their work ability in order to be eligible for benefits from the sickness insurance system, and it is possible to receive sickness benefits covering 25%, 50%, 75%, or 100% of the working time. Swedish employers have broadly legislated responsibility to rehabilitate and adapt work to the worker in order to allow RTW, and part-time sick leave is encouraged by the Swedish social security agency as part of this adaptation [29]. FIXA.

Workplace social capital is important for health, and may have importance for successful RTW from long-term sick leave. However, there are few prospective studies on the impact of social capital in the workplace and how this condition can facilitate RTW and increase vitality among women on long-term sick leave. Therefore, social support-bonding, sense of community-bridging and quality of leadership-linking aspects of social capital are included in previous study. The aim of this study was to investigate the associations between aspects of social capital (social support, sense of community, and quality of leadership) at the workplace, and work ability, working degree, and vitality among women with a history of long-term sick leave from human service organizations.

## Methods

### Research design

A longitudinal cohort study was conducted among women with a history of long-term sick leave working within one of Sweden’s three largest municipalities. The study started in 2005, and the women were followed up at 6 months, 1 year, and 6 years. The study was conducted in accordance with the Declaration of Helsinki [30], and the project was approved by the Ethics Committee of Gothenburg Region at Gothenburg University, Sweden (2005 and 2012).

### Sample

The inclusion criteria at the start of the recruitment time for this cohort were: being a woman, being employed by the municipality, and being on long-term (>60 days) sick leave (ranging from 100% sick leave to at least a part-time sick leave degree of 50%) [31]. In 2005, the employer identified 633 individuals working within the municipality (employees) fulfilling these inclusion criteria. All individuals were sent information about the study via postal mail, and those who replied and agreed to participate received the baseline questionnaire. Individuals answering the baseline questionnaire (*n* = 324) were mailed follow-up questionnaires at 6 months, 12 months, and 6 years, with non-respondents being sent one reminder letter. The response rate was 72% (*n* = 233) at 6 months, 60% (*n* = 194) at 1 year, and 57% (*n* = 185) at 6 years. Earlier studies have been reported from this cohort [20, 31, 32]. To be included in the present study, individuals had to be in employment at the 6-year follow-up (not necessarily with the same employer). The study population thus included 283 individuals from the baseline cohort, 192 from the 6-month follow-up, 153 individuals from the 1-year follow-up, and 144 from the 6-year follow-up.

### Outcome variables

All variables used were individuals’ self-reported data from the questionnaires at baseline, 6 months, 12 months, and 6 years. *Work ability* was assessed with a single item from the Work Ability Index measuring work ability: “How is your current work ability, compared to your lifetime best work ability?”, responded to on a scale from 0 to 10. This single-item score is known as the Work Ability Score (WAS), and has been validated and found to be sensitive for prospective data [29, 31]. The score can be classified into four categories of work ability: poor (0–5), moderate (6–7), good (8–9), and excellent (10) [33]. *Working degree* was used as a measure of RTW, assessed with items covering current working status (change from full-time to part-time sick leave or from part-time sick leave to full-time working). Working degree ranged from 0% to 100%. *Vitality* was measured using an index from the Copenhagen Psychosocial Questionnaire (COPSOC) including four items: “Did you feel full of pep?”, “Did you have a lot of energy?”, “Did you feel worn out?”, and “Did you feel tired?”. In order for all response options to be in the same direction, the two last options were turned. Responses were given on a five-point scale and then recalculated to 0–100 points, with 100 indicating maximum vitality.

### Independent variables

The COPSOQ instrument, which is valid and reliable [34], was used to measure the psychosocial environment of the workplace. Items chosen for this study were social support, sense of community, and quality of leadership. *Social support* was measured by combining two items from COPSOC to reflect the overall support at the workplace, both from superiors and colleagues: “How often do you get help and support from your immediate superior?” and “How often do you get help and support from your colleagues?”. Responses were given on a five-point scale, recalculated to 0–100 points by summing the scores from the two items and dividing by 2, and dichotomized into often (>50) or rarely (≤50) [34]. According to COPSOC, 50 is a neutral value and is natural to use as cut off. *Sense of community* was measured using one item from COPSOC: “Is there good co-operation between your colleagues at work?”. Responses were given on a five-point scale, recalculated to 0–100 points, and dichotomized into high (>50) or low (≤50). *Quality of leadership* was also measured using two items from COPSOC: “To what extent would you say that your immediate superiors are good at solving conflicts?” and “To what extent would you say that your immediate superiors give high priority to job satisfaction?”. Responses were given on a five-point scale, recalculated to a total of 0–100 points, and thereafter categorized into good/high (≥75), moderate (74–26), and poor/low (≤25).

### Data analysis

Descriptive statistics of proportions were calculated for the variables of interest. Next, repeated mixed models were used for longitudinal analysis of the repeated measurements of prospective degree of WAS, working degree, and vitality. These analyses were performed with three different models, with the explanatory variables being social support, sense of community, or quality of leadership and time (baseline, 6 months, 1 year, and 6 years). The outcome variables WAS, working degree, and vitality were assumed to be continuous, and the data for assessment were assumed to be normally distributed. All least squares means analyses were statistically significant at *p* ≤ 0.001. Data were analysed using version 11 of the JMP software package (SAS Institute Inc., Cary, NC, USA).

## Results

Around 50% (*n* = 133) of the study group were between 45 and 55 years of age at baseline (Table 1). Almost one-third (*n* = 85) were working within elderly care/home care, two-fifths as teachers in schools (*n* = 55) or preschools (*n* = 53), and the remainder in administration (*n* = 44), social care (*n* = 16), disability care (*n* = 23), and other sectors (*n* = 51). Almost half (46%) had a university degree. One-third (32%) had both a musculoskeletal disorder and a mental health disorder. At baseline, the mean working degree was 20%, the mean WAS was 3.9 (indicating poor work ability), and the mean vitality score was 40. The mean working degree rose to 50% at 6 months, 60% at 1 year, and 72% at 6 years. Throughout this period of time all the women had been going in and out of work participation [35]. The mean WAS remained poor at 6 months and 12 months (5.1 and 5.5, respectively), but became moderate (6.7) at 6 years. Vitality scores were similar, starting at 40 at baseline and rising to 44 at 6 months, 45 at 1 year, and 49 at 6 years.

**Table 1:** Descriptive statistics at baseline for women with a history of long-term sick leave

Variable	Description	*n* (%)
Age group	35–44 years	84 (30)
	45–54 years	133 (48)
	≥55 years	63 (22)
Disease/disorder	Musculoskeletal	131
*(individuals could have more than one disease, and so percentages are not applicable)*	Mental health	116
	Cardiac	17
	Pulmonary	25
	Comorbidity musculoskeletal & mental health	90
Education	Primary school	49 (18)
	Secondary school	99 (36)
	University	128 (46)
Occupation	Elderly care/home care	85 (29)
	Preschool teacher	53 (18)
	Disability care	23 (8)
	School teacher	55 (19)
	Administration	44 (15)
	Other	35 (12)
Outcome variables		Mean (SD)
Work Ability Score (WAS)	(0–10)	3.9 (2.8)
Working degree (%)	(0–100)	20 (35)
Vitality score	(0–100)	40.2 (21.3)

*n* numbers

### Social support, sense of community, and quality of leadership

Women who reported that their immediate superiors gave a high priority to employees’ job satisfaction increased their WAS significantly more over time (*p* ≤ 0.001) and also scored higher in comparison to those who reported that their immediate superiors gave a low priority to job satisfaction (Table 2). The same pattern could be seen for individuals who reported that their immediate superiors were good at solving conflicts. Individuals reporting a high sense of community increased their WAS significantly more over time and also scored higher in comparison to those reporting a low sense of community. Participants who stated that they often felt they had help and support from immediate superiors and colleagues increased their WAS significantly more over time and also scored higher in comparison to those who did not. Additionally, women whose immediate superiors gave high priority to job satisfaction increased their working degree significantly more over time and also had higher working degree. Individuals who reported their immediate superiors were good at solving conflicts reported higher working degree on all occasions. Women who felt a sense of community at the workplace increased their working degree significantly more over time (*p* ≤ 0.001), and also had a higher working degree (Table 3). Women reporting good quality of leadership, in terms of immediate superiors who were good at solving conflicts and gave high priority to job satisfaction, reported higher vitality at all times in comparison to women who reported poor quality of leadership (Table 4).

**Table 2:** Mixed models repeated measures of Work Ability Score (WAS) among women with a history of long-term sick leave

Models/Explanatory Variables	Work Ability Score (1–10)				Difference between groups 1 and 2	Difference between groups 2 and 3
					Over time	Over time
	LSM (SE)^a^	LSM (SE)^a^	LSM (SE)^a^	LSM (SE)^a^	Estimate (SE)	Estimate (SE)
*Groups*	Baseline	6 months	1 year	6 years	*p*-value	*p*-value
Social support						
*Help and support from immediate superiors and colleagues*						
*1. Often*	4.5 (0.2)	5.4 (0.3)	5.2 (0.3)	6.3 (0.2)	2.2 (0.2)	na
*2. Rarely*	4.1 (0.2)	4.8 (0.2)	4.6 (0.2)	5.8 (0.2)	<0.001	
Sense of community						
*Co-operation between colleagues*						
*1. High*	4.5 (0.2)	5.3 (0.2)	5.2 (0.2)	6.3 (0.2)	2.6 (0.3)	na
*2. Low*	3.7 (0.3)	4.4 (0.3	4.1 (0.4)	5.2 (0.3)	<0.001	
Quality of leadership						
*Immediate superiors good at solving conflicts*						
*1. Good*	6.4 (0.7)	6.4 (0.9)	6.9 (0.8)	7.3 (0.8)	4.1 (0.9)	2.8 (0.4)
*2. Moderate*	4.3 (0.2)	5.3 (0.2)	5.1 (0.2)	6.0 (0.2)	<0.001	<0.001
*3. Poor*	3.2 (0.3)	3.5 (0.4)	3.7 (0.4)	5.3 (0.4)		
Immediate superiors give high priority to job satisfaction						
*1. High*	4.3 (0.5)	6.1 (0.6)	6.1 (0.6)	8.6 (0.6)	*5.3 (0.7)*	2.6 (0.4)
*2. Moderate*	4.4 (0.2)	5.2 (0.2)	5.0 (0.2)	5.9 (0.2)	*<0.001*	<0.001
*3. Low*	3.3 (0.4)	3.5 (0.4)	3.5 (0.4)	5.1 (0.4)		

^a^Least squares mean (standard error)

*na* not applicable

**Table 3:** Mixed models repeated measures of working degree among women with a history of long-term sick leave

Models/Explanatory Variables	Working degree (0–100%)				Difference between groups 1 and 2	Difference between groups 2 and 3
					Over time	Over time
	LSM (SE)^a^	LSM (SE)^a^	LSM (SE)^a^	LSM (SE)^a^	Estimate (SE)	Estimate (SE)
*Groups*	Baseline	6 months	1 year	6 years	*p*-value	*p*-value
Social support						
*Help and support from immediate superiors and colleagues*						
*1. Often*	26.7 (3.5)	51.5 (4.0)	57.3 (4.1)	62.2 (3.7)	41.3 (4.9)	na
*2. Rarely*	21.0 (3.2)	41.3 (3.5)	45.1 (4.0)	60.5 (3.6)	<0.001	
Sense of community						
*Co-operation between colleagues*						
*1. High*	23.5 (2.8)	48.3 (3.1)	54.5 (3.3)	66.4 (3.1)	45.6 (5.3)	na
*2. Low*	20.8 (4.3)	38.9 (5.0)	39.8 (5.9)	48.6 (4.7)	<0.001	
Quality of leadership						
*Immediate superiors good at solving conflicts*						
*1. Good*	57.7 (10.2)	52.8 (12.5)	69.2 (13.7)	76.4 (13.6)	61.4 (14.7)	44.1 (6.3)
*2. Moderate*	22.4 (2.8)	47.9 (3.1)	52.8 (3.3)	59.1 (3.0)	<0.001)	<0.001
*3. Poor*	15.0 (5.6)	36.0 (6.4)	38.3 (6.7)	69.6 (6.1)		
*Immediate superiors give high priority to job satisfaction*						
*1. High*	22.7 (8.6)	41.5 (9.2)	54.0 (9.2)	98.6 (10.1)	82.6 (11.8)	43.1 (6.7)
*2. Moderate*	23.7 (2.7)	48.3 (3.1)	53.5 (3.3)	59.1 (2.9)	<0.001	<0.001
*3. Low*	16.0 (6.0)	33.8 (6.2)	34.5 (6.7)	63.0 (6.3)		

^a^Least squares mean (standard error)

*na* not applicable

**Table 4:** Mixed models repeated measures of vitality among women with a history of long-term sick leave

Models/Explanatory Variables	Vitality (0–100)				Difference between groups 1 and 2	Difference between groups 2 and 3
					Over time	Over time
	LSM (SE)^a^	LSM (SE)^a^	LSM (SE)^a^	LSM (SE)^a^	Estimate (SE)	Estimate (SE)
*Groups*	Baseline	6 months	1 year	6 years	*p-value*	*p-value*
Social support						
*Help and support from immediate superiors and colleagues*						
*1. Often*	47.4 (1.7)	47.4 (2.1)	47.1 (2.1)	54.5 (2.2)	15.8 (2.7)	na
*2. Rarely*	38.7 (1.6)	41.4 (2.0)	43.9 (2.1)	47.5 (2.1)	<0.001	
Sense of community						
*Co-operation between colleagues*						
*1. High*	46.5 (1.4)	46.2 (1.6)	47.2 (1.6)	49.8 (1.7)	17.7 (2.7)	na
*2. Low*	32.0 (2.2)	40.2 (2.6)	38.0 (2.9)	52.0 (2.5)	<0.001	
Quality of leadership						
*Immediate superiors good at solving conflicts*						
*1. Good*	55.0 (5.3)	47.6 (7.0)	54.7 (7.0)	53.8 (6.4)	19.3 (7.0)	18.3 (3.4)
*2. Moderate*	42.2 (1.4)	45.7 (1.6)	44.5 (1.7)	52.8 (1.7)	0.006	<0.001
*3. Poor*	34.5 (2.9)	36.4 (3.4)	39.8 (3.5)	38.3 (3.1)		
*Immediate superiors give high priority to job satisfaction*						
*1. High*	46.4 (4.2)	44.7 (5.0)	54.2 (4.8)	56.0 (5.3)	23.6 (6.2)	20.3 (3.6)
*2. Moderate*	43.5 (1.4)	46.3 (1.6)	44.0 (1.7)	52.7 (1.7)	<0.001	<0.001
*3. Low*	32.4 (3.1)	34.5 (3.3)	41.3 (3.5)	38.4 (3.5)		

^a^Least squares mean (standard error)

*na* not applicable

## Discussion

This study demonstrates the importance of social capital at work, both for the RTW process and for the experience of vitality. The main results show that, over time, social support at the workplace and quality of leadership significantly increased work ability, working degree, and vitality among women on long-term sick leave. Additionally, a sense of community at the workplace significantly increased the women’s WAS and working degree. Earlier research has also shown that supportive conditions at the workplace, such as quality of leadership, sense of community, meaning at work, possibilities for development, and feeling welcomed back to work, play an important role in a sustainable RTW process [20, 36–43].

The results of this study reveal that a sense of community over time had significant importance for working degree at the 6-year follow-up. Healthy working relationships and having high social support from coworkers are both promoting factors for a sustainable RTW [44]. This is in line with previous findings that people on long-term sick leave rated interaction with others and belonging to a group at the workplace as the most supportive factors in relation to work performance, satisfaction, wellbeing [15], and having a feeling of being welcomed back to work [20].

The duration of sickness absence can be considerably reduced if the contact between the sick-listed individual and the workplace occurs at an early stage [45]. A systematic literature review showed that early multidisciplinary interventions and time-bound plans of action appear most effective to support RTW [37]. One way to achieve this is to actively and strategically involve the entire team when planning a sick-listed individual’s RTW. Being on long-term sick leave involves feelings of not being a part of a social context, not being needed, and having no ability to make a difference for others [16, 46, 47]. Although our study shows that a high sense of community between coworkers promotes RTW, other studies have revealed concerns among coworkers that their workload will increase if they are expected to support their colleague’s RTW process [38, 48, 49].

Being on sick leave can be difficult in a society that values independence and being capable, strong, healthy, and productive. Such a society does not favour the sense of community which could promote an early RTW among women on long-term sick leave. Moreover, interviews with sick-listed individuals have revealed descriptions of increased egocentrism in the workplace, jealousy, and a lack of solidarity and more negative attitudes towards the sick-listed person [16]. Women on sick leave experience feelings of being torn between what they want to be and what they can manage, their ideals having become more like demands, and being expected to both be and appear to be a capable and independent individual despite their circumstances [50].

Our study reveals that both RTW and the experience of vitality significantly increased if the women’s immediate superiors gave high priority to job satisfaction at the workplace. The results additionally show that immediate superiors who were good at solving conflicts contributed to earlier RTW and vitality among the women. A previous study from the same cohort also showed the importance of leadership quality in relation to WAS over time [20]. However, another cohort study found no associations between quality of leadership and RTW after long-term sickness absence among eldercare staff [51]. Thus, aspects of the importance of leadership for RTW must be further studied.

The workplace is an important social arena, and many people find their identity through work. It is at work that many important social relationships are created and maintained [36]. Work is also a prerequisite for maintaining the same living standards as before sick leave. This highlights the importance of promoting an early RTW. There is great interest in developing strategies to promote a sustainable RTW from a personal, social, and economic perspective.

One beneficial approach is to take the woman’s life-world into account and to see her life situation as a whole, rather than just focusing on the limitations that the disease can bring. With such an approach, the individual’s strengths can be utilized and help the individual to meet the challenges that life may bring. A holistic approach can be facilitated through teamwork between medical experts, superiors, colleagues, and other actors involved, all of whom contribute their specific expertise.

There are reasons to believe that such a procedure is easier to implement in large workplaces. In smaller workplaces, there is a risk that colleagues will have to take greater responsibility for the work that would be implemented by the woman on long-term sick leave. However, for a sustainable RTW, it is important that the above aspects are taken into account regardless of the size of the workplace.

### Strengths and limitations

One strength of this particular study is the prospective nature when investigating the importance of social capital in many aspects, i.e. in terms of quality of leadership, sense of community and social support at the workplace. Another is the use of mixed models when analysing repeated measurements over time. The study also have a number of weaknesses which may imply some limitation in the interpretation of the findings. First, the relative common challenge with low response rate at baseline (51%) but more important for interpretation were the losses at the follow-up measures (72%, 60% and 57%). Our analyses showed that the non-respondents to the follow-up questionnaires were fairly equally distributed over the age groups (chi-square, >0.0001) and there was no significant difference between non-respondents to the follow-up questionnaires and respondents regarding how the main variable (social capital) was answered at baseline. Further, the method for analyses (repeated measurements) allows the data to be smartly clustered for each individual. When using mixed models for longitudinal analyses of repeated measurements, it is less important to estimate missing data, as the available data will be used if subjects are missing [52]. In the present study, the data were collected on several occasions, and we cannot say anything about what happened between the different measurements. This must also be considered as a limitation of the study. Furthermore, the longer people are on sick leave, the greater is the risk that they will not return to work [53, 54]. In a Swedish cohort study it was found that stable high level of sickness absence among employees was associated with estimated low social support at work [55]. However, these results are not completely comparable; as the studies are based on different inclusion criteria, you might assume that people with history of long term sick leave in a higher degree rate their work environment as less supportive.

Finally, there are variables which may have been confounding factors, such as education, civil status, and age. However, the sample in this study can be seen as homogenous in that the participants had the same gender and the same employer, and all age groups were represented.

## Conclusions

A sustainable return-to-work process among individuals with a history of long-term sick leave, going in and out of work participation, could be supported with social support, good quality of leadership, and a sense of community at the workplace. The responsibility for the rehabilitation process can not be reduced to an individual problem, but ought to include all the stakeholders involved in the process, such as managers, colleagues, health care services, and the social security agency.
